# Use of Artificial Intelligence in Cobb Angle Measurement for Scoliosis: Retrospective Reliability and Accuracy Study of a Mobile App

**DOI:** 10.2196/50631

**Published:** 2024-11-01

**Authors:** Haodong Li, Chuang Qian, Weili Yan, Dong Fu, Yiming Zheng, Zhiqiang Zhang, Junrong Meng, Dahui Wang

**Affiliations:** 1 Department of Orthopedics Children’s Hospital of Fudan University National Children’s Medical Center Shanghai China

**Keywords:** scoliosis, photogrammetry, artificial intelligence, deep learning

## Abstract

**Background:**

Scoliosis is a spinal deformity in which one or more spinal segments bend to the side or show vertebral rotation. Some artificial intelligence (AI) apps have already been developed for measuring the Cobb angle in patients with scoliosis. These apps still require doctors to perform certain measurements, which can lead to interobserver variability. The AI app (cobbAngle pro) in this study will eliminate the need for doctor measurements, achieving complete automation.

**Objective:**

We aimed to evaluate the reliability and accuracy of our new AI app that is based on deep learning to automatically measure the Cobb angle in patients with scoliosis.

**Methods:**

A retrospective analysis was conducted on the clinical data of children with scoliosis who were treated at the Pediatric Orthopedics Department of the Children’s Hospital affiliated with Fudan University from July 2019 to July 2022. Three measurers used the Picture Archiving and Communication System (PACS) to measure the coronal main curve Cobb angle in 802 full-length anteroposterior and lateral spine X-rays of 601 children with scoliosis, and recorded the results of each measurement. After an interval of 2 weeks, the mobile AI app was used to remeasure the Cobb angle once. The Cobb angle measurements from the PACS were used as the reference standard, and the accuracy of the Cobb angle measurements by the app was analyzed through the Bland-Altman test. The intraclass correlation coefficient (ICC) was used to compare the repeatability within measurers and the consistency between measurers.

**Results:**

Among 601 children with scoliosis, 89 were male and 512 were female (age range: 10-17 years), and 802 full-length spinal X-rays were analyzed. Two functionalities of the app (photography and photo upload) were compared with the PACS for measuring the Cobb angle. The consistency was found to be excellent. The average absolute errors of the Cobb angle measured by the photography and upload methods were 2.00 and 2.08, respectively. Using a clinical allowance maximum error of 5°, the 95% limits of agreement (LoAs) for Cobb angle measurements by the photography and upload methods were –4.7° to 4.9° and –4.9° to 4.9°, respectively. For the photography and upload methods, the 95% LoAs for measuring Cobb angles were –4.3° to 4.6° and –4.4° to 4.7°, respectively, in mild scoliosis patients; –4.9° to 5.2° and –5.1° to 5.1°, respectively, in moderate scoliosis patients; and –5.2° to 5.0° and –6.0° to 4.8°, respectively, in severe scoliosis patients. The Cobb angle measured by the 3 observers twice before and after using the photography method had good repeatability (*P*<.001). The consistency between the observers was excellent (*P*<.001).

**Conclusions:**

The new AI platform is accurate and repeatable in the automatic measurement of the Cobb angle of the main curvature in patients with scoliosis.

## Introduction

Scoliosis is identified as a spinal deformity characterized by lateral curvature and vertebral rotation, diagnosed through an X-ray indicating a Cobb angle greater than 10° [1]. The incidence rate of scoliosis in children is between 1% and 3% [2]. The causes of scoliosis are diverse and cannot be prevented [3]. Early screening during adolescence is crucial for identifying scoliosis, where most cases are mild but can progress rapidly in 10% to 20% of patients, necessitating regular quantitative monitoring [4]. Regular and quantitative monitoring is essential for these patients.

At present, the measurement of the Cobb angle is still the most commonly used quantitative index to evaluate scoliosis severity. It has important reference value for the diagnosis, choice of treatment strategy, and evaluation of the curative effect of scoliosis [5]. However, in clinical practice, Cobb angle measurement is different within and between observers [6,7]. Manual Cobb angle measurement has 3-5° of intraobserver variation and 5-7° of interobserver variation [8]. However, these values are reported for experienced raters, and thus, the variation could be higher for less experienced raters. This error may be caused by the different selection of the end vertebrae and the manual error during measurement [4]. When the child’s positioning is not standardized during the radiography session, the quality of the full-length spinal X-ray may be compromised, failing to accurately reflect the true extent of the child’s spinal curvature and the size of the Cobb angle. This is because the human spine itself has a certain degree of mobility. For instance, if the child’s upper limbs are positioned asymmetrically during the X-ray, it will directly cause the spine to bend. This results in the measured Cobb angle being either larger or smaller compared to the child’s actual Cobb angle, further leading to errors in the measurement of the Cobb angle. After these variations are compounded, some clinicians may have a measurement error of 5-10° in assessing the Cobb angle of a child, which is sufficient to lead clinicians to misjudge the progression of a patient’s scoliosis, thereby causing the child to miss the optimal treatment opportunity [7,9].

Various methods for measuring the Cobb angle exist, ranging from traditional manual measurements with a pencil and protractor on X-ray films to digital methods like the Picture Archiving and Communication System (PACS) used increasingly in hospitals [10,11]. Despite advancements, challenges persist in tool specificity, usage conditions, and measurement repeatability.

In recent years, artificial intelligence (AI) models have been shown to be remarkably successful in the interpretation of medical images [12]. Due to advances in the computing power of smartphones, health care professionals can easily obtain AI-generated measurement data through a user-friendly mobile app system to assist in the diagnosis, prediction, and treatment of diseases. The mobile app can intelligently recognize and capture images for the assessment of body posture and measurement of the Cobb angle in kyphosis, delivering accurate results. Moreira et al [13] compared the NLMeasurer with a validated biophotogrammetry software for 6 posture measurements and found that the NLMeasurer, a mobile app based on the PoseNet deep learning algorithm, provides highly reliable measurements for frontal posture assessment.

In the realm of image-based mobile apps, the 2 commonly used smartphone apps for measuring the Cobb angle in scoliosis are the iPinPoint and Cobbmeter apps, which are available for download from the Apple iTunes store. Numerous researchers have investigated these apps [11,14,15]. The iPinPoint and Cobbmeter apps are mobile apps that have integrated spinal X-ray capture using a smartphone and an interface that allows evaluators to manually mark the upper and lower vertebrae of the full-length spinal X-ray using touchscreen functionality.

Despite the significant advancements that these mobile apps for measuring the Cobb angle represent in the scoliosis assessment process, desktop solutions still require the manual identification of the upper and lower vertebrae. After capturing the spinal X-ray with a mobile device, the examiner must mark the planes of the endplates of the terminal vertebrae within the software interface on the phone before the app can measure the Cobb angle. This process may lead to variability in the results, depending on the examiner’s ability to accurately identify the terminal vertebrae.

The application of AI in radiology holds enormous potential in achieving more accurate diagnosis of diseases and improving treatment decision-making. Using the deep learning of AI, we developed an app (cobbAngle pro) that could automatically measure the Cobb angle of scoliosis. This app, designed for mobile devices, is user-friendly, requires minimal technical expertise in imaging and scene setup, and eliminates the need for the examiner to manually select the terminal vertebrae to measure the Cobb angle. This functionality offers flexibility during the assessment process and reduces the occurrence of human errors when selecting the terminal vertebrae.

This study attempts to evaluate the reliability of the new AI phone app based on deep learning.

## Methods

### General Information

We conducted a retrospective study on patients with scoliosis who came to our center from July 2019 to July 2022. The patients’ spinal X-rays were all sourced from the PACS. The inclusion criteria were as follows: (1) age 10-18 years; (2) diagnosis of noncongenital scoliosis; (3) ability to complete photography in a standard posture (The patient stands with feet slightly apart, knees and hips naturally extended, torso without external force interference, both upper limbs naturally hanging down, leaning on the support poles on both sides of the torso to reduce the impact of the upper limbs on the torso’s balance, chin lifted, and eyes looking straight ahead).

The exclusion criteria were as follows: (1) congenital scoliosis and secondary scoliosis (unequal length of lower limbs, etc) and (2) nonstandard shooting positions (This refers to a posture that does not adhere to standard radiographic techniques. Common nonstandard postures for full-length anterior-posterior spinal radiographs primarily include asymmetrical positioning of the upper limbs on both sides and 1 lower limb positioned in front of the other).

### Principles of Intelligent Application

**Figure 1:**
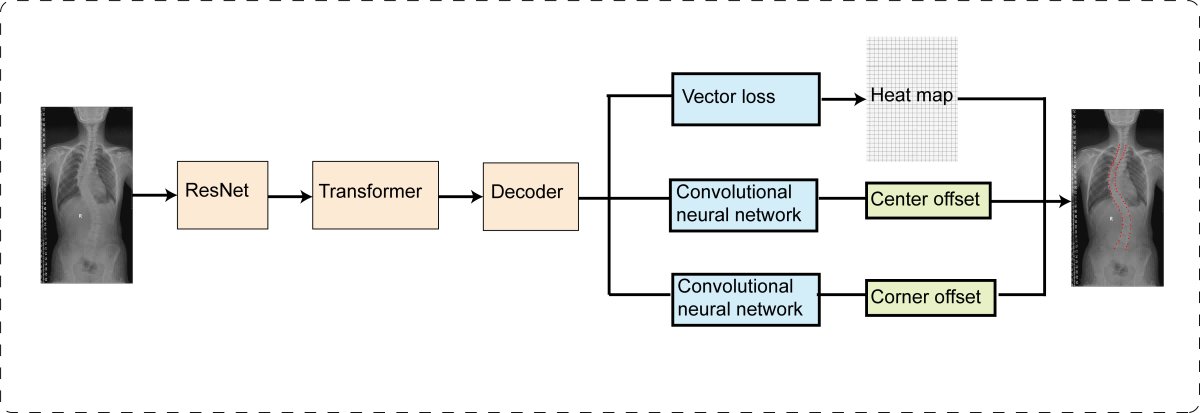
Network framework.

In this study, an AI app was developed to automatically calculate the Cobb angle from spinal X-rays using deep learning and image processing techniques. The model employed by this mobile app uses a neural network framework based on the transformer mechanism, which directly predicts the Cobb angle and vertebral coordinates from the preprocessed training dataset. The neural network architecture comprises an encoder, a decoder, and a transformer module. The encoder is based on the ResNet network, with the final fully connected layer removed. The extracted feature blocks are passed to the transformer module. In this module, the feature maps extracted by the convolutional neural network are unfolded into 1-dimensional features, which are then combined with positional encoding. The transformer’s inherent encoder and decoder extract spatial correlations, resulting in the output of 1-dimensional features. These features are subsequently converted into tensor blocks and fed into the decoder. The transformer module is essential for adjusting the dimensionality between the input and output layers, as well as for performing data denoising. The decoder receives the tensor blocks and queries feature blocks of the same size as those obtained during the encoding stage. It then performs deconvolution operations to ultimately generate a heatmap of the central point coordinates and offset vectors. This module is used to annotate the central points in the images, thereby enhancing the network’s recognition efficiency. Finally, an angle offset loss function is incorporated into the network to ensure that the training results converge toward accurate predictions. The following text outlines the data processing procedures, AI model training procedures, and principles for confirming the Cobb angle using the AI model (Figure 1).

### Data Collection

We collected 20,000 spinal X-rays from various hospitals. For each X-ray, 4 points on all vertebrae were manually annotated to ensure that each image was paired with the corresponding Cobb angle annotation.

### Preprocessing

The images underwent preprocessing steps, such as normalization, grayscaling, and scaling, to meet the input requirements of ResNet. Data augmentation techniques (eg, rotation, scaling, and cropping) were applied to enhance data diversity and prevent model overfitting.

### Data Splitting

The current dataset consists of 18,000 images for training and 2000 images for testing, with a 9:1 ratio. During the training process, 9-fold cross-validation was employed to mitigate overfitting. Based on hospital statistics, the ratio of mild-to-moderate-to-severe cases among pediatric orthopedic outpatients was 5:5:2. However, in the first phase of data collection, the ratio of mild-to-moderate-to-severe cases was 9:6:1. This does not align with the true distribution of severity levels, as there is a shortage of severe cases, leading to an imbalance in the dataset. To address this issue, we collected additional imaging data, including X-rays, from severe pediatric cases. During the training process, we also removed some data from mild cases to alleviate the data imbalance and better approximate the true distribution observed in our pediatric hospital.

### Model Inference

Finally, a pretrained ResNet variant deep learning model was applied to the preprocessed images for feature extraction and key point detection, identifying the 4 key points on all vertebrae.

The program uses the moving least squares (MLS) method for curve fitting to obtain the overall shape of the spine. Polynomial fitting is then used to calculate the second derivative of the curve. Changes in the second derivative reflect the curvature and inflection points of the curve, indicating changes in the bending direction. Based on the bending direction, the spinal curve is divided into multiple segments to calculate the Cobb angle for each segment. For each segment, 2 lines are fitted to the upper and lower edges of the vertebrae using key points, and a loop iterates through all possible line combinations to calculate the intersection angles. Angles below 5° are ignored.

The method for calculating the Cobb angle for each line combination is as follows:

First, the tangent of the angle between the 2 lines is calculated using the following formula:



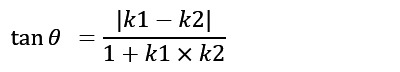



Then, the angle is calculated using the arctangent function:



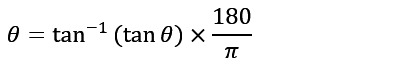



Among all the calculated angles, the largest Cobb angle is selected as the Cobb angle for the corresponding segment. After calculating the Cobb angles for all segments, the 2 largest Cobb angles are identified, with the slightly larger angle referred to as the major angle and the slightly smaller angle referred to as the minor angle.

### Model Fairness and Code Privacy

Our trained model has demonstrated good fairness, as the calculation method for spinal vertebrae does not exhibit differences based on factors, such as gender, making the model broadly applicable. Due to data privacy concerns, however, we have not publicly shared the trained model or the code.

### Research Methods

#### Selection of Observers

We selected 3 fellow doctors in pediatric orthopedics (with at least 2 years of working experience in spinal deformities) as observers. The developers equipped the 3 observers with the same type of terminal equipment and conducted mobile app operation training until they could accurately and skillfully use the app to measure the Cobb angle. When measuring the Cobb angle for scoliosis using the mobile app, it is essential to ensure that the photographed scene includes all spinal segments in the X-ray, covering the entire length of the spine. This is crucial for accurately measuring the primary Cobb angle of scoliosis patients. The plane of the photo should also be as parallel to the X-ray film as possible to avoid significant measurement errors due to improper use of the app method. Therefore, training for the 3 observers was necessary.

#### Study Setting

This study was conducted in the orthopedic doctors’ office of the first ward of the inpatient department at the Children’s Hospital of Fudan University. Three observers performed manual measurements using 3 PACS-equipped computers in the office. The process of measuring the Cobb angle with the mobile app was also carried out at the same location.

### Measurement Methods

The X-ray films of children were disordered, and the identification information of each film was covered up. The person who selected the film and the observers were completely unaware of the patients’ information. To reduce the impact of subjective and informational biases, the measurers first measured the main curvature Cobb angle using the PACS method, followed by measurements with the mobile app, with a specific time interval (2 weeks) between the 2 measurement methods. After measuring the main curvature Cobb angle with the PACS method, the measurers waited 2 weeks before continuing to measure the main curvature Cobb angle of the same set of spinal X-rays of patients using the mobile app by the photography and photo upload methods, further eliminating the impact of information bias. The measurement data were recorded on a separate data sheet. After 2 weeks, 3 observers took the second measurement to reduce their own deviation.

#### PACS Method

Three observers used the measuring tools of the PACS to determine the upper and lower vertebrae of the main curvature of scoliosis on the computer, marked the upper and lower endplates of the upper and lower vertebrae, and then measured their Cobb angles with the measuring tools and recorded the data.

#### Photography Method

**Figure 2:**
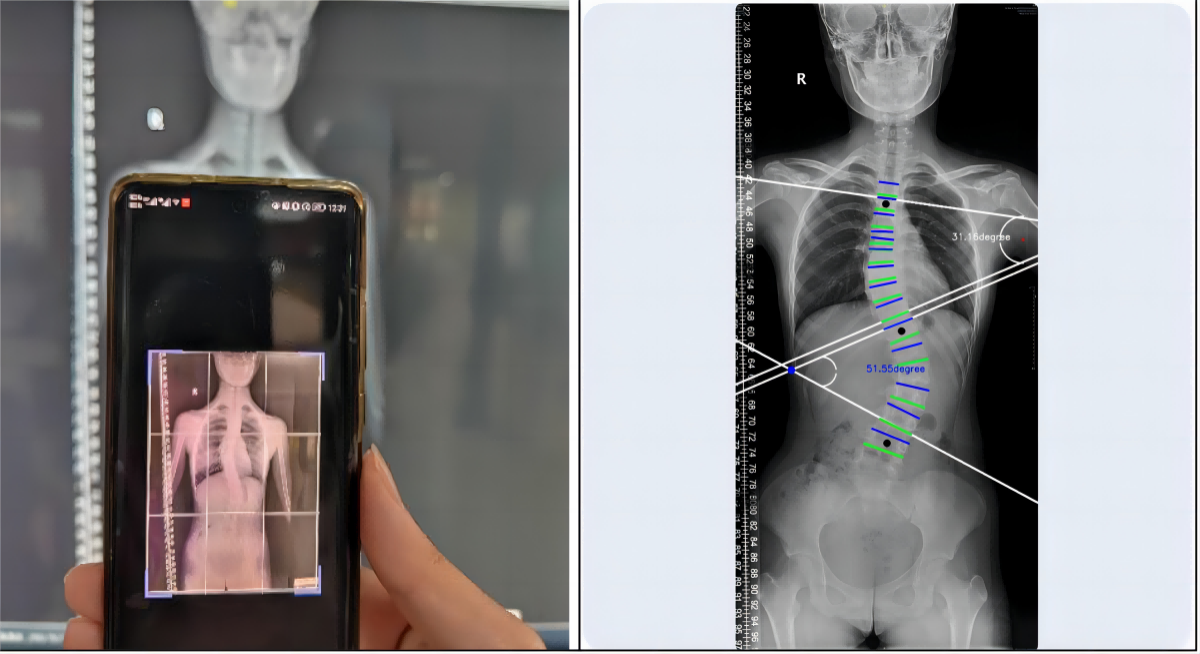
Cobb angle measured by the photography method.

In order to reduce subjective bias, the time of measuring the Cobb angle by using the 2 functions of the app was determined after the PACS method was completed. When shooting photos of the X-ray film on the computer, the full length of the spine was included in the shooting interface, and the mobile phone posture was parallel to the position of the X-ray film as much as possible, and was aligned and kept stable to reduce errors. After the successful shooting, the software automatically recognized and displayed the Cobb angle, and the 3 measurers recorded it individually (Figure 2).

#### Photo Upload Method

**Figure 3:**
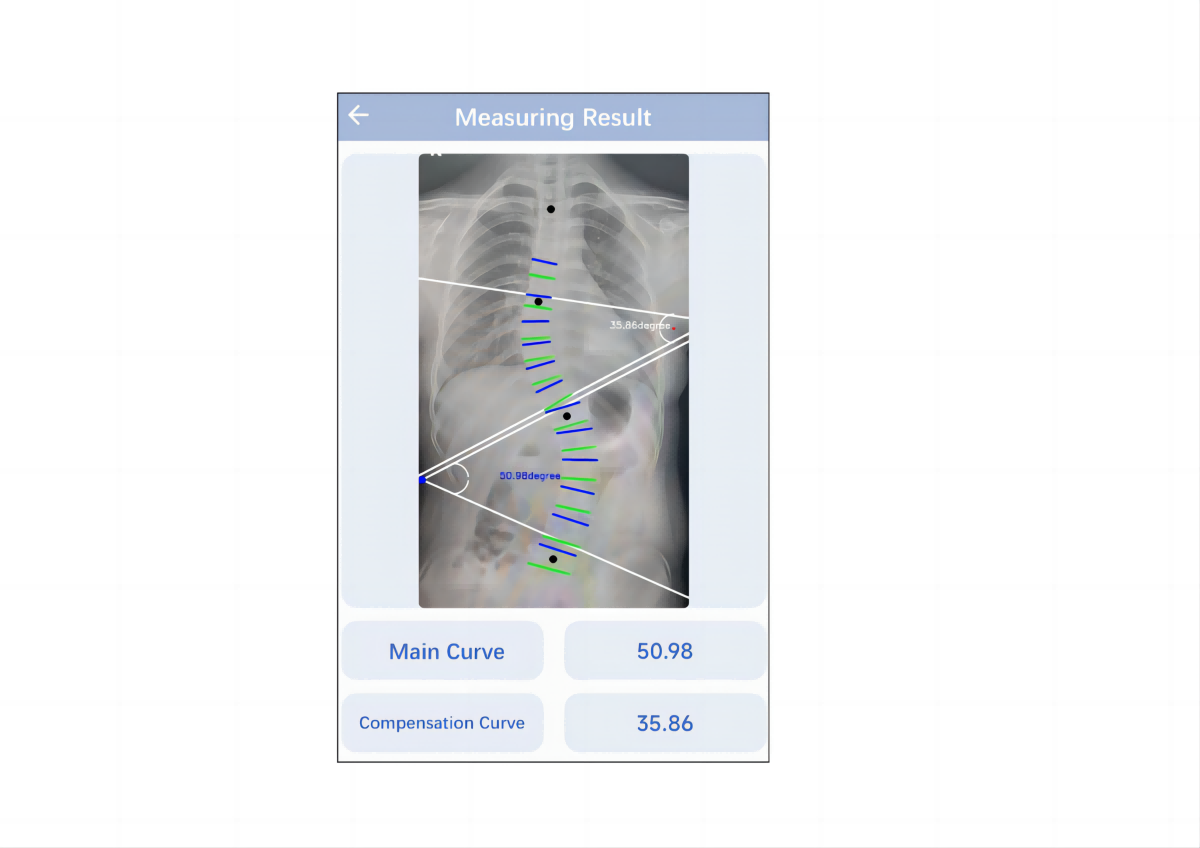
Cobb angle measured by the photo upload method.

We downloaded the X-ray films of all the included samples from the hospital system and saved them in the photo album of the test phone. Subsequently, we selected the “photo upload” function of the app to upload the original X-ray images of all samples. The app will automatically recognize the image and measure its Cobb angle. The data were recorded by 3 observers (Figure 3).

### Statistical Analysis

Software SPSS v20 (IBM Corp) and MedCalc (MedCalc Software Ltd) were used for statistical analysis. The intraclass correlation coefficient (ICC) was used to compare the Cobb angle measurement results of the app method (photography and photo upload) and PACS method to judge the consistency of the measurement results. For ICC, 0.250-0.499 indicates poor consistency, 0.500-0.699 indicates moderate consistency, 0.700-0.899 indicates good consistency, and ≥0.900 indicates excellent consistency. A *P*-value <.05 was considered to indicate a statistically significant difference.

We divided the subjects into 3 groups according to the degree of scoliosis (mild: Cobb <25°, moderate: 25° ≤ Cobb ≤ 45°, severe: Cobb >45°) and used the Bland-Altman method to test the consistency of the Cobb angle measurement between the app and the PACS within the groups. The Bland-Altman test was used to obtain the 95% limits of agreement (LoAs) based on the average difference of ±1.96 SD between the 2 measurement methods. The measurement error of the 2 methods has been represented by drawing a scatter diagram.

We used the ICC for both reliability analysis (interobserver difference analysis) and repeatability analysis (intraobserver difference analysis). In these assessments, for ICC, 0.250-0.499 indicates poor consistency, 0.500-0.699 indicates moderate consistency, 0.700-0.899 indicates good consistency, and ICC ≥0.900 indicates excellent consistency. A *P*-value <.05 was considered to indicate a statistically significant difference.

### Ethical Considerations

#### Human Subject Ethics Review Approvals

This study was approved by the Medical Ethics Committee of the Children’s Hospital of Fudan University (reference number: 2019-184). The content and process of this study comply with the ethical requirements for biomedical research issued by international and national authorities.

#### Informed Consent Privacy and Confidentiality

The children included in the study or their guardians signed the informed consent form. In the initial informed consent form, participants were thoroughly informed that this study would involve secondary analysis of the Cobb angle data obtained from the collected spinal X-rays.

#### Privacy and Confidentiality

In the initial informed consent form, it was explicitly stated that the study would anonymize personal information, such as name, gender, birth date, and ID, when measuring the Cobb angle data from spinal X-rays. Access to the final summary tables containing original data with patient identifiers was restricted, allowing only authorized research team members to access sensitive information. At the outset of the study, clear data protection policies and standard operating procedures were established to ensure that all researchers are aware of and comply with data protection regulations.

## Results

### General Information

This study included 601 patients (aged from 10 to 17 years; mean 12.75, SD 1.54 years). Of these 601 patients, 89 were male (14.8%) and 512 were female (85.2%). Among the 802 full-length orthopedic films, 383 showed mild severity, 349 showed moderate severity, and 70 showed severe severity. The results of manually measuring the Cobb angle using the PACS method are shown in Table 1.

**Table 1:** Manual measurement of the Cobb angle by the Picture Archiving and Communication System (PACS) method.

Severity^a^	Cobb angle (°)	
	Mean (SD)	Maximum
Mild (n=383)	19.43 (3.35)	24.92
Moderate (n=349)	32.99 (5.52)	44.93
Severe (n=70)	52.04 (6.51)	75.02

^a^Mild: Cobb <25°, moderate: 25° ≤ Cobb ≤ 45°, and severe: Cobb >45°.

### Intra- and Interobserver Consistency Test

The ICC values for the 3 measurers using the PACS method before and after 2 measurements were all greater than 0.9, indicating very good intra- and interobserver consistency. The ICC results for the 2 measurements using the mobile app method are shown in Table 2. Both methods demonstrated excellent intra- and interobserver consistency. The intraobserver ICC and interobserver ICC using the mobile photography method were superior to those of the PACS method.

**Table 2:** Intra- and interobserver agreement analysis.

Variable	PACS^a^ method, ICC^b^ (95% CI)	Photography method, ICC (95% CI)	*P* value
Intraobserver 1	0.989 (0.987-0.990)	0.996 (0.995-0.996)	<.001
Intraobserver 2	0.983 (0.981-0.986)	0.996 (0.995-0.997)	<.001
Intraobserver 3	0.984 (0.981-0.986)	0.996 (0.996-0.997)	<.001
Interobserver	0.992 (0.991-0.993)	0.997 (0.997-0.998)	<.001

^a^PACS: Picture Archiving and Communication System.

^b^ICC: intraclass correlation coefficient.

### Reliability Analysis of the AI App

Based on the excellent interobserver and intraobserver consistency of the PACS method, we used the PACS method as a reference to evaluate the reliability of the AI app measurement capability. We compared 2 app measurements (photography and photo upload) with the PACS method for measuring Cobb angles, and evaluated the results using ICC tests, as shown in Table 3. In this test, the ICC values for the comparison of the photography method and PACS method by 3 observers were greater than 0.9 and statistically significant (*P*<.001). Similarly, the ICC values for the photo upload method and PACS method were greater than 0.9 and statistically significant (*P*<.001). This indicated that there is good consistency between the app measurement and manual measurement results, suggesting that these 2 methods of measuring the Cobb angle are equivalent.

**Table 3:** Consistency analysis of measurement results between applied measurement methods and the Picture Archiving and Communication System (PACS) method.

Observer	PACS^a^ method, mean (SD)	Photography method			Photo upload method	
		Mean (SD)	ICC^b^	Mean (SD)		ICC
Observer 1	28.16 (10.90)	28.03 (11.21)	0.973	28.14 (11.33)		0.972
Observer 2	28.16 (10.93)	28.05 (11.22)	0.971	28.14 (11.33)		0.973
Observer 3	28.20 (10.95)	28.02 (11.18)	0.971	28.14 (11.33)		0.972

^a^PACS: Picture Archiving and Communication System.

^b^ICC: intraclass correlation coefficient.

**Figure 4:**
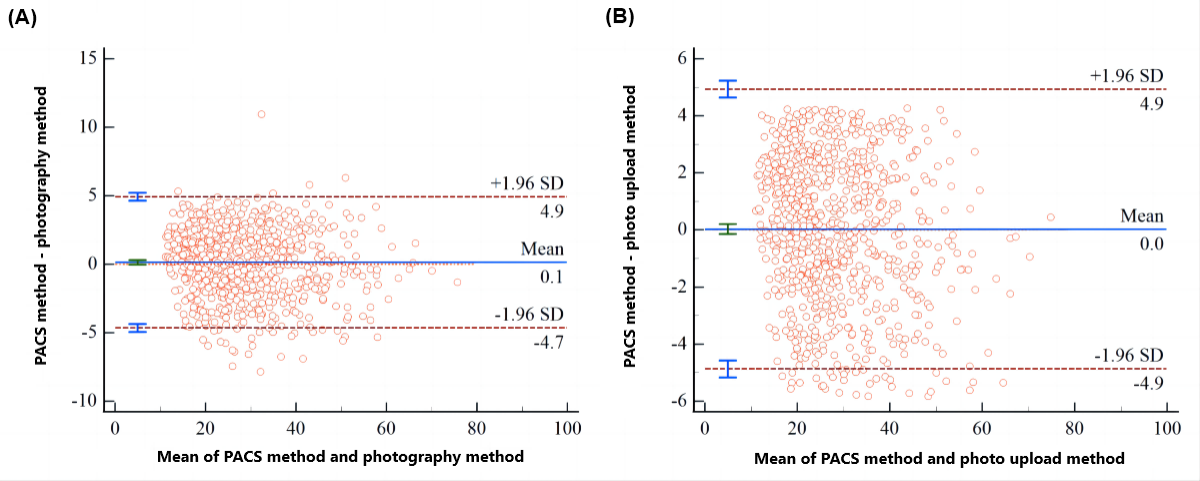
The mean signed differences between the Picture Archiving and Communication System (PACS) method and the photography method (A) and photo upload method (B).

When measuring the Cobb angle using the photography and photo upload methods, the mean absolute errors were very small (all less than 5°) compared to the PACS method, as shown in Table 4. It is generally considered that an increase of more than 5° in the Cobb angle between 2 consecutive X-ray examinations indicates the progression of scoliosis. Clinically, the allowable measurement error for the Cobb angle is not to exceed 5° [16]. Thus, we defined a measurement error of <5° as “accurate” and took the measurement results of the PACS method as the reference standard. As shown in Table 5, the smartphone app demonstrated a high accuracy rate in measuring the Cobb angle. The relationship between the mean signed differences of the 2 app methods and the PACS method, as shown in Figure 4, indicated that for all subjects, the 95% LoAs for the differences were all less than 5°.

**Table 4:** Cobb angle error of the main curvature from the smartphone app.

Function and severity		Error	
		Mean (SD)	Maximum
**Photography**		2.00 (1.43)	10.96
	Mild (n=383)	1.87 (1.33)	7.43
	Moderate (n=349)	2.12 (1.47)	10.96
	Severe (n=70)	2.00 (1.64)	6.33
**Photo upload**		2.08 (1.38)	5.83
	Mild (n=383)	1.93 (1.32)	5.57
	Moderate (n=349)	2.21 (1.39)	5.83
	Severe (n=70)	2.27 (1.63)	5.83

**Table 5:** Accuracy analysis of the smartphone app.

Function and severity			Error of <5°, n		Accuracy rate, %	
**Photography**						
	Mild (n=383)	375		97.9		
	Moderate (n=349)	339		97.1		
	Severe (n=70)	65		92.9		
**Photo upload**						
	Mild (n=383)	373		97.4		
	Moderate (n=349)	337		96.6		
	Severe (n=70)	63		90.0		

**Figure 5:**
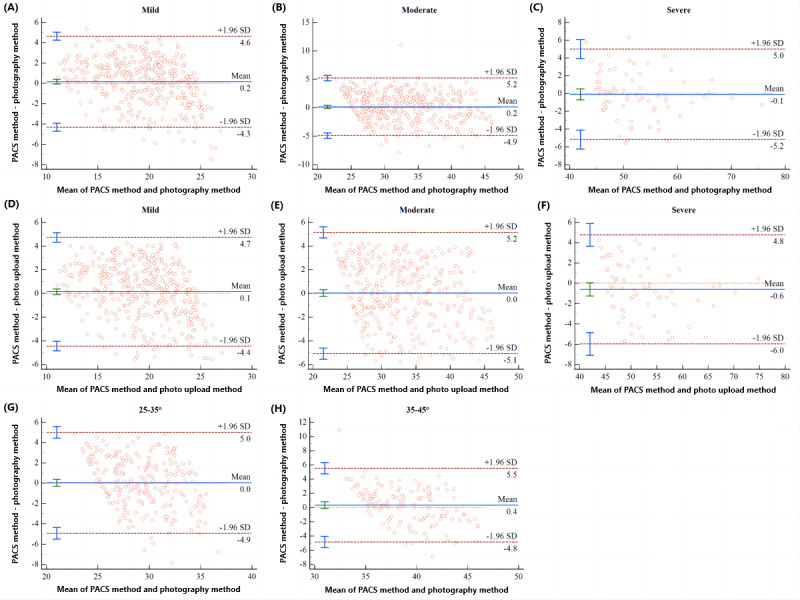
The mean signed differences between the Picture Archiving and Communication System (PACS) method and photography method for mild scoliosis (A), moderate scoliosis (B), and severe scoliosis (C). The mean signed differences between the PACS method and photo upload method for mild scoliosis (D), moderate scoliosis (E), and severe scoliosis (F). The mean signed differences between the photography method and PACS method for moderate scoliosis with Cobb angles between 25° and 35° (G), and between 35° and 45° (H).

We categorized subjects into 3 subgroups based on the severity of spinal curvature in our study. The mean signed differences and 95% LoAs between the PACS method and photography method across the 3 subgroups are depicted in Figure 5A-C. The outcomes for the mean signed differences and 95% LoAs between the PACS method and photo upload method in these subgroups are presented in Figure 5D-F. Lastly, we focused on subjects with Cobb angles between 25° and 35° for manual measurement and analyzed the mean signed difference between the photography method and PACS method. The 95% LoAs for the difference were precisely –5° to 5°, as shown in Figure 5G. This indicates that AI-based app measurements of the Cobb angle in patients with mild scoliosis are more reliable, and the app measurements could be more accurate for patients with scoliosis of less than 35°.

### Endplate Selection Variability

Observer 1 used the PACS method to select the upper and lower end vertebrae on 670 full-length anteroposterior and lateral spine X-rays of 542 children and recorded the results. After an interval of 2 weeks, the end vertebrae were selected and recorded again using the mobile photo album upload function, comparing the consistency of end vertebrae selection. It is important to note that the mobile app does not directly provide the selection results of the upper and lower end vertebrae. Instead, it displays 2 white extension lines representing the major curve angle. The spinal segments that these white lines pass through are manually recorded and considered as the upper and lower end vertebrae determined by the mobile app’s algorithm. Table 6 provides details of the difference in upper and lower endplate selection between successive pairs of phone upload method and PACS method measurements. As shown in Table 6, the selection of end vertebrae was not completely consistent between the 2 measurement methods (46.9% agreement in upper endplate selection vs 57.8% agreement in lower endplate selection).

**Figure 6:**
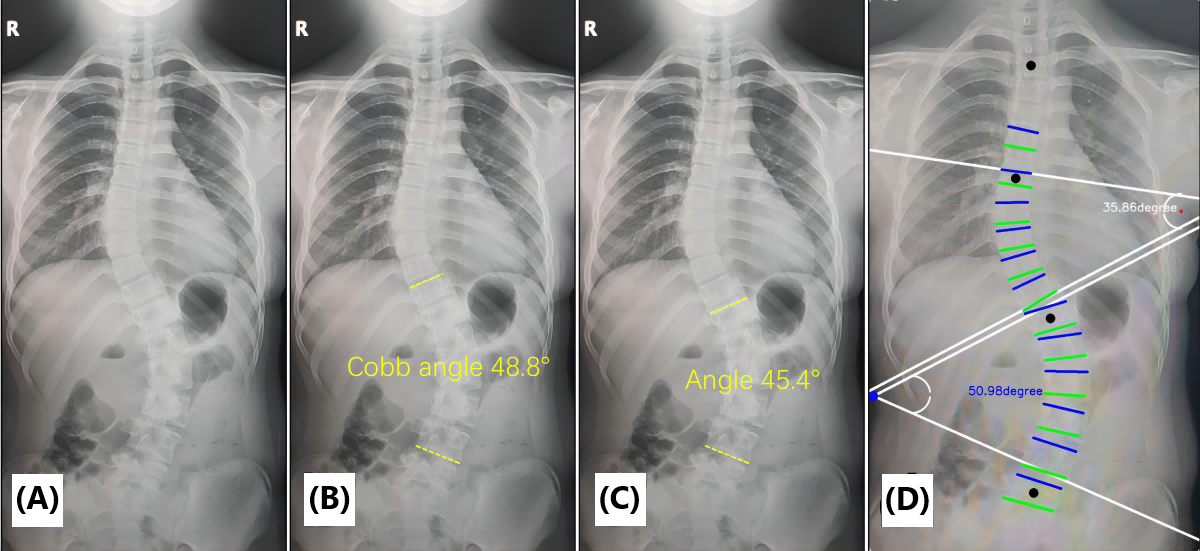
Lenke 5 scoliosis (A), with the upper and lower end vertebrae measured by the Picture Archiving and Communication System (PACS) method being T11 and L4, respectively (B). (C, D) The scoliosis curve depicted by the photo upload method shows that the maximum scoliosis angle is between T12 and L4.

Figure 6 shows the measurement of end vertebrae for a Lenke 5–type scoliosis patient using 2 methods, which revealed that the upper vertebral selection of the app method was 1 level lower than that of the PACS method (T11 to L4 for the PACS method vs T12 to L4 for the photo upload method).

**Table 6:** Comparison of differences in spinal end selection between the photo upload method and Picture Archiving and Communication System (PACS) method by observer 1.

Vertebral selection		Value (N=670), n (%)
**Upper vertebral selection**		
	The upper vertebral selection of the app method is 1 level higher than that of the PACS^a^ method	62 (9.3)
	Select the same end vertebra level	314 (46.9)
	The upper vertebral selection of the app method is 1 level lower than that of the PACS method	294 (43.9)
**Lower vertebral selection**		
	The lower vertebral selection of the app method is 2 levels higher than that of the PACS method	11 (1.6)
	The lower vertebral selection of the app method is 1 level higher than that of the PACS method	81 (12.1)
	Select the same end vertebra level	387 (57.8)
	The lower vertebral selection of the app method is 1 level lower than that of the PACS method	179 (26.7)
	The lower vertebral selection of the app method is 2 levels lower than that of the PACS method	12 (1.8)

^a^PACS: Picture Archiving and Communication System.

## Discussion

### Principal Findings

Our AI mobile app for measuring the Cobb angle can accurately and automatically mark the coordinates of the apex for each vertebra, fit the spinal curvature, and perform Cobb angle measurements. Evaluators only need to check that the mobile capture function of the software includes a complete X-ray image of the child’s spine. Additionally, this AI mobile app automatically calculates the Cobb angle for children with scoliosis and marks the end vertebrae. Analysis of the Cobb angle measurement results indicates that this mobile app shows effectiveness when compared to the PACS.

Carman et al [9] pointed out that intraobserver variability is an important parameter in the clinical diagnosis and treatment of scoliosis, as intraobserver differences may lead to misdiagnosis of curve progression, thereby impacting clinical treatment decisions. However, Shaw et al [11] emphasized that interobserver consistency is equally important when measuring the Cobb angle, as in many cases the same patient may not be measured by the same physician. In this study, for the mobile app, the intraobserver ICC was 0.996 and the interobserver ICC was 0.997, both of which exceeded the values for the PACS method. The mobile app demonstrated significantly better intraobserver and interobserver reproducibility when measuring the Cobb angle. Good consistency can effectively prevent medical errors caused by measurement inaccuracies.

Compared to the PACS method, the mobile app method showed significantly better intra- and interobserver repeatability in measuring the Cobb angle. Good consistency can effectively avoid medical errors caused by measurement errors in patients.

In order to explore the accuracy of the software measurement, we took the Cobb angle results obtained by the PACS method as a reference and compared the differences between the photography and upload methods and the PACS method. We found that the errors in the Cobb angle measured by the photography and upload methods and the PACS method were 2.00 and 2.08, respectively. The accuracy of the mobile phone app photography function in measuring patients with mild and moderate scoliosis was greater than 97%. Compared with the PACS method, the mean differences in the Cobb angle measured by the photography and upload methods were 0.1° and 0.03°, respectively, and the 95% consistency limits were –4.7° to 4.9° and –4.9° to 4.9°, respectively, which are less than the allowable error range (5°) [10]. Thus, the Cobb angle measured by the mobile phone app can be considered to be reliable compared with the traditional PACS manual measurement.

However, we believe that the current accuracy of the app will decrease with the severity of the patient’s scoliosis. The results of subgroup analysis showed that there was a difference of >5° between mild and moderate severity scoliosis. We assume that the critical value of the Cobb angle with this difference may be in patients with moderate scoliosis. In order to find the critical value for the app, we attempted to use the photography method to measure the X-ray film of patients with a Cobb angle ≤35°, and the results showed that the 95% consistency limit was just –5° to 5°, which is in line with the maximum measurement error range allowed by clinical practice. The results suggest that the AI app is more accurate in measuring patients with scoliosis with a Cobb angle ≤35°. The reason for this is likely the insufficient deep learning data for X-ray images of severe scoliosis, and further data input is needed. It just fits the applicable population of the software: patients with scoliosis who need close and regular follow-up.

### Comparison With Prior Work

We reviewed the intraobserver and interobserver variability and errors of other smart apps for measuring the Cobb angle, such as CobbMeter and Tiltmeter software. For CobbMeter, the intraobserver ICC was 0.983 and interobserver ICC was 0.973 [14]. In contrast, both the intraobserver and interobserver ICC for our app were greater than 0.99, indicating that the software program in this study has more considerable repeatability in measuring the Cobb angle compared to the CobbMeter app. The Tiltmeter software had an average absolute error of 2.1° (SD 1.7°) in measuring the Cobb angle, while our mobile app had an error of only 2.0° (SD 1.43°), showing that our mobile app has smaller errors in measuring the Cobb angle compared to similar apps and demonstrating superior performance in measuring the Cobb angle among apps of its kind [10].

Similar to this, the mobile intelligent app NLMeasurer uses a PoseNet solution based on computer vision and machine learning for human posture assessment and anatomical point identification. Research by Moreira et al [13] demonstrated that posture measurements calculated using NLMeasurer were consistent with validated biophotogrammetry software SAPO, proving it to be an effective tool for evaluating posture measurements in the frontal view. Consistent with our study’s conclusions, these mobile apps show superior reliability and convenience in measuring body posture angles or Cobb angles for scoliosis compared to traditional computer-based methods like the PACS or SAPO. Additionally, the NLMeasurer app exhibited high interobserver and intraobserver reliability similar to our AI Cobb angle measurement app.

However, unlike the NLMeasurer mobile app, our developed AI app for measuring the Cobb angle has a significant advantage in that it requires no manual identification or annotation of key anatomical landmarks (end vertebrae) on the captured images to accurately measure the Cobb angle. While the NLMeasurer app can also perform posture measurements directly on unmarked photos, it benefits from using surface markers on specific anatomical landmarks (such as the ears, iliac crests, and ankle joints) to facilitate digital recognition of these points, thereby improving the reliability of posture measurements performed with NLMeasurer. Therefore, to enhance the accuracy of posture assessment using photos without surface anatomical markers, the NLMeasurer app would still need to optimize its related algorithms.

### Strengths and Limitations

#### The AI Mobile App Can Fully Automate Cobb Angle Measurement, Reducing Errors Associated With Manually Marking End Vertebrae

With the rapid advancement of computer technology, computer-aided methods for measuring the Cobb angle have been widely applied in clinical practice and have proven to have reliability, good correlation, and measurement accuracy compared to traditional manual measurements [17-19]. Digital computer-aided measurement methods, such as Surgimap software [20] and the PACS, exemplify this trend. With the popularity of smartphones, researchers have developed numerous mobile apps for measuring the Cobb angle. These apps, such as Scoligauge, Tiltmeter, and CobbMeter, are considered to be more reliable and convenient for screening and measuring the Cobb angle in cases of scoliosis or kyphosis compared to other methods [11,21,22].

However, all the aforementioned methods require the examiner to manually identify and mark the upper and lower end vertebrae of the scoliosis patient before measuring the Cobb angle, which means the measurement results may be biased by the examiner’s ability to accurately identify the end vertebrae. In contrast, our AI mobile app truly achieves fully automated Cobb angle measurement, allowing users to take a photo or upload an image to measure the Cobb angle without needing to premark the end vertebrae. This functionality provides flexibility during the assessment process and reduces the likelihood of human errors when placing markers.

#### The AI Mobile App Offers Flexible Multiscenario Usage Compared to Large Computers

The PACS has not yet been universally adopted in all hospitals, which limits the ability of clinical physicians to use it in certain situations. When clinicians consult with patients outside the hospital, the computer-based PACS cannot be accessed. If patients bring soft or hard copies of digital X-rays taken at other hospitals, doctors cannot retrieve the imaging data from their hospitals’ computers, forcing them to either manually measure the Cobb angle or have the patient undergo repeat X-rays at their facility. The former reduces result reliability, while the latter increases the patient’s radiation risk and costs. In these scenarios, the mobile app can be used flexibly, allowing clinicians to measure the Cobb angle in children with scoliosis without needing a computer. When patients wish to have online consultations, they can send their spinal X-ray images via the internet, enabling doctors to upload and measure the Cobb angle directly using the app, thus assisting clinicians in determining whether the patient’s scoliosis has progressed. When patients consult with X-rays taken at outside facilities, they do not need to undergo repeat imaging, and clinicians can quickly take pictures and measure the Cobb angle using the software.

#### The Use of the AI Mobile App in Guiding the Formulation of Surgical Strategies Holds Significant Potential Value

When measuring patients with moderate to severe spinal curvature, some discrepancies still exist between the results obtained by the app and the PACS. These differences may arise partly from insufficient data for deep learning, and we suspect that the algorithm itself also plays a role. Unlike manual Cobb angle measurements, the app does not first identify the upper and lower vertebrae of the scoliosis. Instead, it uses AI to plot the spinal curve in the coronal plane and subsequently determines the maximum angle of curvature on that curve. Therefore, we believe that the Cobb angle measured by the app is more accurate than that obtained via the PACS method.

To address these discrepancies, we reanalyzed the relationship between the segment of the Cobb angle line depicted by the app and the upper and lower vertebrae identified by the PACS method. Our findings indicated that, in some instances, the upper and lower vertebrae determined by the 2 methods differed by 1 to 2 vertebral bodies (Table 6). In cases of idiopathic scoliosis, this definition directly influences the surgical strategy for correcting spinal curvature, highlighting the significance of an app that can measure vertebrae more accurately for patients (Figure 6). The introduction of this app may help preserve the patient’s range of motion by potentially avoiding the need to address the last segment of the spine during surgical planning. In future studies, we will explore orthotic correction and surgical treatment of patients based on the vertebrae and Cobb angle measurements provided by the app.

### Limitations

Despite the encouraging results obtained from the use of the AI Cobb angle measurement app, it is essential to highlight the limitations of this study. First, when the app identifies the primary curve for measurement, it also provides a Cobb angle measurement for a compensatory curve. We need follow-up experiments to validate the app’s reliability in measuring smaller compensatory curves. Second, we believe that there are still errors when measuring the Cobb angle with this app. However, since the app is based on deep learning algorithms, its accuracy can continuously improve. During the data model generation process, more images of abnormal spines can be incorporated, allowing the network to train on various boundary cases, thereby providing a traceable basis for testing.

Additionally, a Fourier transform module can be added to the transformer module to reduce computational load and enhance efficiency through the combination of the 2 modules. Furthermore, based on the practical situations encountered during the use of this model, qualified experts could provide direct feedback on the angles determined by the machine via the human-computer interface. This feedback could serve as part of the model’s penalty function, further improving the model’s accuracy and iterative optimization efficiency. We believe that through the deep learning capabilities of AI, the measurement accuracy of the app will continue to improve in the future.

### Conclusion

This newly developed AI platform mobile app measures the main curvature Cobb angle with reliable results and high accuracy, which can provide convenience for clinicians and is worth promoting in clinical application.

## Abbreviations

AI: artificial intelligence
ICC: intraclass correlation coefficient
LoAs: limits of agreement
PACS: Picture Archiving and Communication System

## Data Availability

Data will be made available upon reasonable request.

## References

[1] Altaf F, Gibson A, Dannawi Z, Noordeen H (2013). Adolescent idiopathic scoliosis. BMJ.

[2] Weinstein SL, Dolan LA, Cheng JC, Danielsson A, Morcuende JA (2008). Adolescent idiopathic scoliosis. The Lancet.

[3] Peng Y, Wang S, Qiu G, Zhang J, Zhuang Q (2020). Research progress on the etiology and pathogenesis of adolescent idiopathic scoliosis. Chin Med J (Engl).

[4] Jin C, Wang S, Yang G, Li E, Liang Z (2022). A review of the methods on Cobb angle measurements for spinal curvature. Sensors.

[5] Romano M, Minozzi S, Bettany-Saltikov J, Zaina F, Chockalingam N, Kotwicki T, Maier-Hennes A, Negrini S (2012). Exercises for adolescent idiopathic scoliosis. Cochrane Database Syst Rev.

[6] Kuklo TR, Potter BK, Polly DW, O’Brien MF, Schroeder TM, Lenke LG (2005). Reliability analysis for manual adolescent idiopathic scoliosis measurements. Spine.

[7] Morrissy RT, Goldsmith GS, Hall EC, Kehl D, Cowie GH (1990). Measurement of the Cobb angle on radiographs of patients who have scoliosis. Evaluation of intrinsic error. The Journal of Bone & Joint Surgery.

[8] Wong J, Reformat M, Parent E, Stampe K, Southon Hryniuk S, Lou E (2023). Validation of an artificial intelligence-based method to automate Cobb angle measurement on spinal radiographs of children with adolescent idiopathic scoliosis. Eur J Phys Rehabil Med.

[9] Carman DL, Browne RH, Birch JG (1990). Measurement of scoliosis and kyphosis radiographs. Intraobserver and interobserver variation. J Bone Joint Surg Am.

[10] Langensiepen S, Semler O, Sobottke R, Fricke O, Franklin J, Schönau E, Eysel P (2013). Measuring procedures to determine the Cobb angle in idiopathic scoliosis: a systematic review. Eur Spine J.

[11] Shaw M, Adam CJ, Izatt MT, Licina P, Askin GN (2011). Use of the iPhone for Cobb angle measurement in scoliosis. Eur Spine J.

[12] Rajpurkar P, Chen E, Banerjee O, Topol EJ (2022). AI in health and medicine. Nat Med.

[13] Moreira R, Fialho R, Teles AS, Bordalo V, Vasconcelos SS, Gouveia GPDM, Bastos VH, Teixeira S (2022). A computer vision-based mobile tool for assessing human posture: A validation study. Computer Methods and Programs in Biomedicine.

[14] Qiao J, Liu Z, Xu L, Wu T, Zheng X, Zhu Z, Zhu F, Qian B, Qiu Y (2012). Reliability analysis of a smartphone-aided measurement method for the Cobb angle of scoliosis. J Spinal Disord Tech.

[15] Ketenci İE, Yanık HS, Erdoğan Ö, Adıyeke L, Erdem Ş (2021). Reliability of 2 smartphone applications for Cobb angle measurement in scoliosis. Clin Orthop Surg.

[16] Hughes J, Yaszay B, Bastrom T, Bartley C, Parent S, Cahill P, Lonner B, Shah S, Samdani A, Newton P, Harms Study Group (2021). Long-term patient perception following surgery for adolescent idiopathic scoliosis if dissatisfied at 2-year follow-up. Spine (Phila Pa 1976).

[17] Kuklo TR, Potter BK, Schroeder TM, O'Brien M (2006). Comparison of manual and digital measurements in adolescent idiopathic scoliosis. Spine (Phila Pa 1976).

[18] Wills BPD, Auerbach JD, Zhu X, Caird MS, Horn BD, Flynn JM, Drummond DS, Dormans JP, Ecker ML (2007). Comparison of Cobb angle measurement of scoliosis radiographs with preselected end vertebrae: traditional versus digital acquisition. Spine (Phila Pa 1976).

[19] Rosenfeldt MP, Harding IJ, Hauptfleisch JT, Fairbank JT (2005). A comparison of traditional protractor versus Oxford Cobbometer radiographic measurement: intraobserver measurement variability for Cobb angles. Spine (Phila Pa 1976).

[20] Akbar M, Terran J, Ames CP, Lafage V, Schwab F (2013). Use of Surgimap Spine in sagittal plane analysis, osteotomy planning, and correction calculation. Neurosurg Clin N Am.

[21] Franko O, Bray C, Newton P (2012). Validation of a scoliometer smartphone app to assess scoliosis. J Pediatr Orthop.

[22] Jacquot F, Charpentier A, Khelifi S, Gastambide D, Rigal R, Sautet A (2012). Measuring the Cobb angle with the iPhone in kyphoses: a reliability study. Int Orthop.

